# Long-term effectiveness, safety and immunogenicity of the biosimilar SB2 in inflammatory bowel disease patients after switching from originator infliximab

**DOI:** 10.1177/1756284820982802

**Published:** 2021-01-14

**Authors:** Sarah Fischer, Sarah Cohnen, Entcho Klenske, Heike Schmitt, Francesco Vitali, Simon Hirschmann, Andreas Ramming, Sebastian Zundler, Timo Rath, Sabine Krebs, Frank Dörje, Wolfgang Uter, Daniel Nagore, Sebastian Meyer, Markus F. Neurath, Raja Atreya

**Affiliations:** Department of Medicine 1, Friedrich-Alexander-Universität Erlangen-Nürnberg, Erlangen University Hospital, Erlangen, Germany; Department of Medicine 1, Friedrich-Alexander-Universität Erlangen-Nürnberg, Erlangen University Hospital, Erlangen, Germany; Department of Medicine 1, Friedrich-Alexander-Universität Erlangen-Nürnberg, Erlangen University Hospital, Erlangen, Germany; Department of Medicine 1, Friedrich-Alexander-Universität Erlangen-Nürnberg, Erlangen University Hospital, Erlangen, Germany; Department of Medicine 1, Friedrich-Alexander-Universität Erlangen-Nürnberg, Erlangen University Hospital, Erlangen, Germany; Department of Medicine 1, Friedrich-Alexander-Universität Erlangen-Nürnberg, Erlangen University Hospital, Erlangen, Germany; Department of Medicine 3, Friedrich-Alexander-Universität Erlangen-Nürnberg, Erlangen University Hospital, Erlangen, Germany; Department of Medicine 1, Friedrich-Alexander-Universität Erlangen-Nürnberg, Erlangen University Hospital, Erlangen, Germany; Department of Medicine 1, Friedrich-Alexander-Universität Erlangen-Nürnberg, Erlangen University Hospital, Erlangen, Germany; Pharmacy Department, Friedrich-Alexander-Universität Erlangen-Nürnberg, Erlangen University Hospital, Erlangen, Germany; Pharmacy Department, Friedrich-Alexander-Universität Erlangen-Nürnberg, Erlangen University Hospital, Erlangen, Germany; Department of Medical Informatics, Biometry and Epidemiology, Friedrich-Alexander-Universität Erlangen-Nürnberg, Erlangen, Germany; Progenika Biopharma, Derio, Spain; Department of Medical Informatics, Biometry and Epidemiology, Friedrich-Alexander-Universität Erlangen-Nürnberg, Erlangen, Germany; Department of Medicine 1, Friedrich-Alexander-Universität Erlangen-Nürnberg, Erlangen University Hospital, Erlangen, Germany; Department of Medicine 1, University Hospital Erlangen, Friedrich-Alexander-Universität Erlangen-Nürnberg, Ulmenweg 18, Erlangen, 91054, Germany

**Keywords:** biosimilar, inflammatory bowel diseases, infliximab, SB2, switch

## Abstract

**Background:**

Long-term data on inflammatory bowel disease (IBD) patients switched from originator to biosimilar infliximab SB2 are lacking. The aim of the conducted study was to investigate the effectiveness, immunogenicity and safety of a large prospectively followed-up IBD patient cohort that was entirely switched from originator infliximab to biosimilar SB2 treatment.

**Methods:**

This was a prospective, single-center, longitudinal, observational study describing clinical outcomes in IBD patients, over an 80-week period following switch from originator infliximab to SB2. Primary outcome measures were change of disease activity [Harvey-Bradshaw Index for Crohn’s disease (CD), partial Mayo Score for ulcerative colitis (UC)], C-reactive protein (CRP), infliximab trough levels (TLs), anti-drug antibodies (ADAs) and adverse events.

**Results:**

One hundred and forty-four IBD patients (94 CD, 50 UC), with median duration of 30.5 months’ (range 2–110) treatment with originator infliximab were evaluated. Mean change of disease activity compared with baseline was −0.9 (SD 2.6), –0.4 (2.2) and –0.4 (2.0) in CD; 0.1 (1.1), 0.1 (1.1) and 0.1 (1.3) in UC patients at weeks 24, 48 and 72. Median infliximab TLs were 6.2 µg/ml (interquartile range 2.3–12.2), 5.0 µg/ml (2.7–10.0), 6.6 µg/ml (3.5–12.4) and 5.1 µg/ml (2.7–10.9) at baseline and weeks 24, 48 and 72. Median CRP levels were within normal ranges throughout the study. After the switch, 9.8% of the patients developed new ADAs. Persistence on SB2 was 90% (95% confidence interval 0.85–0.95), 79% (0.72–0.86), 72% (0.64–0.80) at weeks 26, 52 and 78. Serious adverse events occurred in 11 patients.

**Conclusion:**

Over the individual patient follow-up of 80 weeks, switch to biosimilar SB2 from originator infliximab does not result in increased disease activity or changed immunogenicity patterns. The switch to SB2 was well tolerated.

## Introduction

Biological therapies are potent treatment options in various immune mediated inflammatory diseases and have become an integral part of the therapy of inflammatory bowel disease (IBD) patients. The anti-tumor necrosis factor antibody infliximab was the first biologic drug approved for the treatment of IBD in 1998 and its clinical application improved the outcome for a significant subgroup of treated patients.^1^ Due to the rising incidence of IBD and the increasing use of biologic agents, substantial healthcare costs now represent a growing economic burden.^2^ Upon expiry of the patent for originator infliximab, different biosimilars have entered the market. Biosimilars are defined as biological drugs with high similarity to the reference drugs and no meaningful clinical differences in terms of efficacy, quality and safety.^3^ In 2013, the first biosimilar infliximab, CT-P13 (Remsima^®^, Inflectra^®^), was approved for the treatment of IBD patients. Switching IBD patients from ongoing infliximab originator to biosimilar therapy for cost reasons was initially accompanied by concerns regarding possible immunogenic related loss of efficacy, as well as uncertainty around potential safety issues.^4^

Several observational studies indicated that effectiveness, safety and immunogenicity were not compromised in IBD patients switched to CT-P13.^5–9^ The NOR-SWITCH trial, a randomized, comparative study, demonstrated non-inferiority of CT-P13 to the infliximab originator over the course of 52 weeks.^5^ A recently published phase III non-inferiority study enrolled 220 patients with active Crohn’s disease (CD) and randomized them 1:1:1:1 on treatment arms that underwent either infliximab originator or biosimilar CT-P13 induction therapy and were thereafter either maintained on the induction drug or switched to the other one. The primary endpoint (CD activity index –70 response) again demonstrated non-inferiority of biosimilar CT-P13 in comparison with originator infliximab.^10^ Nevertheless, data regarding the infliximab biosimilar SB2 (Flixabi^®^, Renflexis^®^), which was approved by the European Medicines Agency in 2016 and by the Food and Drug Administration in 2017, are rather scarce in the entire field of immune mediated inflammatory disorders. The approval of SB2 was based on a phase I pharmacokinetic study in healthy individuals and phase III study results that indicated equivalent efficacy, safety and immunogenicity outcomes of originator infliximab and SB2 in patients with moderate to severe rheumatoid arthritis.^11–14^ There is one study of 96 chronic plaque psoriasis patients that were switched from CT-P13 to SB2, with a follow-up period of 6 months. The switching procedure was not associated with a significant change in the mean Psoriasis Area and Severity Index.^15^

There are only two reports of SB2 treated IBD patients, which are both derived from an observational cohort study conducted in Italy. In the first published report, 35 SB2 treated IBD patients were investigated for 6 months, with eight (10.6%) of them switched from originator infliximab to SB2, while 85.7% were naïve to infliximab and 3.9% switched from biosimilar CT-P13 to SB2.^16^ Overall, steroid-free remission after week 8 was achieved by 48.6% of the patients, while 22.8% reached partial response. Serious adverse events (SAEs) occurred in seven of 77 patients, with an incidence rate of 49.3 per 100 person-years. A separate assessment, only for the originator infliximab to SB2 switched IBD patients, was not reported in the publication.

The other study publication with a follow-up time of 18 months also included only a small fraction of patients that were switched from originator infliximab to SB2 (17 of 276), as nearly 70% of the analyzed patients were naïve to infliximab.^17^ Assessment of safety was the primary endpoint of the study, with a total follow-up time of 182.7 person-years. Here, an SAE incidence rate of 36.7 per 100 person-years was recorded. Clinical effectiveness at weeks 8 and 52 was only evaluated in the 192 IBD patients naïve to infliximab. At week 8, 57.3% of infliximab naïve IBD patients had steroid-free remission and 13.5% achieved partial response, while of the 74 IBD patients with a 52 weeks follow-up, 24.3% had steroid-free remission and 39.2% achieved a partial response at that time point.^17^ Data regarding immunogenicity of the SB2 treated patients were missing. We here present the first long-term data over an 18-month follow-up period that aimed to assess the effectiveness, immunogenicity and safety of a large prospectively followed-up IBD patient cohort that was entirely switched from originator infliximab to biosimilar SB2 treatment.

## Materials and methods

### Study design

This study was conducted as a single-center, prospective, observational, longitudinal, cohort study at the outpatient Clinic for IBD of the Medical Department 1 of the University Hospital Erlangen, Germany. All patients receiving originator infliximab therapy were switched between February and April 2017 to the biosimilar SB2 for non-medical reasons and followed up for a period of up to 80 weeks. Every patient received careful counseling by a physician before switching to SB2. SB2 was used throughout the study according to the recommended indications and dosages for infliximab, which included intensification of therapy according to the physician’s discretion based on the presented clinical or endoscopic disease activity (shortening of the administration intervals up to every 4 weeks and/or increasing the dose up to 10 mg/kg bodyweight). Patient’s respective trough-level (TL) and anti-drug antibody (ADA) measurements were not shared with the treating physician throughout the study. Use of concomitant IBD-related medication consisting of corticosteroids or immunosuppressants (thiopurines, methotrexate) was documented. Non-serious adverse events and SAEs were recorded at every patient visit. Blood samples were taken before every SB2 administration. Patients gave written informed consent regarding blood sampling before participating in the study. The sample collection was previously approved by the ethical committee and the institutional review board of the Friedrich-Alexander-University of Erlangen-Nürnberg (proposition approval number: 40_16B).

### Patients

All IBD patients treated with originator infliximab at the outpatient Clinic of the University Hospital Erlangen were eligible for inclusion in the study. Inclusion criteria were: a minimum age of 18 years, established diagnosis of CD or ulcerative colitis (UC), completed induction therapy (weeks 0, 2, 6) with originator infliximab prior to the switching procedure. Patients with an ostomy, pouchitis or microscopic colitis treated with infliximab were excluded. To avoid any potential bias, all IBD patients that were switched to SB2 and fulfilled the stated inclusion and exclusion criteria were eligible for participation in the study. Only patients with baseline data at the time of switching to SB2 regarding TL, anti-infliximab antibodies and C-reactive protein (CRP) levels were eligible for analysis of these variables. Patients with missing baseline blood samples were exclusively evaluated for clinical disease activity scores only.

### Baseline characteristics

Demographic and clinical data including age, sex, body-mass index, disease entity and duration, Montreal classification, onset of infliximab treatment, infliximab dosing, infusion interval and exposure to immunosuppressant or steroid medication were recorded.

### Outcome measures

The primary outcome measure of the study was the evaluation of effectiveness of SB2 (change of clinical disease activity) after 24, 48 and 72 weeks. Secondary outcome measures included change of CRP levels, pharmacokinetic profile, immunogenicity, persistence on SB2 therapy over the study period and assessment of safety. All parameters were evaluated at baseline (time-point of switching to SB2; week 0) and at every respective patient visit for SB2 application during the study period of 80 weeks.

#### Disease activity

Disease activity was evaluated using the Harvey–Bradshaw Index (HBI) in CD and the partial Mayo Score (pMS) in UC patients.^18,19^ The clinical disease activity scores were assessed at every patient visit for SB2 administration by the treating physician as part of the standard care procedures at our institution.

#### CRP

CRP levels were measured at every patient visit. A serum CRP threshold of less than 5 mg/l was considered to be normal.

#### Pharmacokinetics and immunogenicity

Infliximab TL and ADA were measured prior to every infliximab biosimilar SB2 administration using Promonitor^®^ tests of Progenika Biopharma (Derio, Spain), an enzyme-linked immunosorbent assay. The serum probes were diluted to 1:500, resulting in a measurable TL range of 0.2–32.0 µg/ml. Probes with higher TL were diluted by 1:3000 to achieve evaluable results. Baseline TLs before the first administration of the biosimilar SB2 referred to originator infliximab, all follow-up TLs referred to SB2. Therapeutic TLs were defined to lie between 3 µg/ml and 7 µg/ml.^20^ For patients who paused infliximab treatment and were re-introduced to the drug during the study phase, TL at the date of re-introduction and during the re-induction period were excluded. TLs during the maintenance period after completed re-initiation were included. ADA positivity was defined as detectable ADAs regardless of the measured levels.

#### Safety

All adverse events (AEs) were assessed throughout the study period. Every life-threatening, potentially disabling condition or one requiring hospitalization was classified as a SAE.

#### Drug acquisition costs

The Pharmacy Department of the University of Erlangen-Nürnberg calculated the selling prices for all applied SB2 vials in our study and compared them with manufacturer’s selling price for the equivalent amount of originator infliximab vials.

### Statistics

Disease activity scores, CRP levels, ADA and TLs were summarized at intervals of 8 weeks. If multiple visits of the same patient fell in the 8-week window, the visit closest to the regular 8-week interval was used for the analysis. We computed descriptive statistics by time point and IBD cohort, using counts and proportions for categorical variables, and the median (interquartile range) for metric variables. Means and standard deviations (SDs) were calculated for the change in clinical disease activity. We used a combination of box and beeswarm plots to visualize the distribution of metric variables, including individual measurements. Therapy persistence was analyzed using the Kaplan–Meier estimator for the time to SB2 discontinuation, where right censoring included loss to follow-up, end of study, and therapy interruptions due to competing events (pregnancy, remission). All statistical analyses were carried out in the statistical software environment R 3.6.2 (R Core Team, 2019).^21^

## Results

### Baseline study population, disease and treatment characteristics

**Figure 1.**
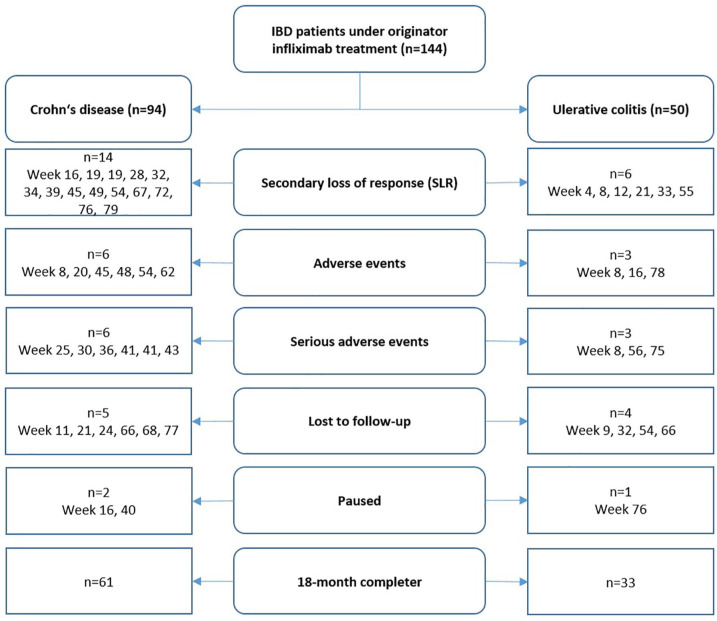
This flowchart describes the study discontinuations and the underlying reasons for all patients enrolled, separated for Crohn’s disease and ulcerative colitis. The time-point of the study discontinuation for the respective reason is indicated. IBD, inflammatory bowel disease.

We enrolled a total of 144 IBD patients (45.8% females), consisting of 94 CD (65.3%) and 50 UC (34.7%) patients (Figure 1). The demographics were well balanced between the disease entities regarding sex, age, age at diagnosis, disease duration, body-mass index and duration of previous infliximab treatment. All baseline characteristics are displayed in detail in Table 1.

**Table 1.** Patient characteristics at baseline.

	Crohn’s disease *n* = 94	Ulcerative colitis *n* = 50	IBD *n* = 144
Sex			
Female	46 (48.9%)	20 (40.0%)	66 (45.8%)
Male	48 (51.1%)	30 (60.0%)	78 (54.2%)
Age, median (range)	39 (19–78)	42.5 (19–68)	39.5 (19–78)
Age at diagnosis, years, median (range)	22 (6–72)	26 (6–61)	24 (6–72)
BMI, kg bodyweight/m^2^, median (range)	24.5 (16.5–50.1)	25.2 (17.5–37.9)	24.8 (16.5–50.1)
Disease duration, years, median (range)	9 (1–50)	8 (2–30)	8 (1–50)
Montreal classification, *n* (%)			
A1	13 (13.8%)	E 1	2 (4.0%)
2	69 (73.4%)	2	18 (36.0%)
3	12 (12.8%)	3	30 (60.0%)
L1	22 (23.4%)		
2	15 (16.0%)		
3	57 (60.6%)		
4	22 (23.4%)		
B1	26 (27.7%)		
2	18 (19.1%)		
3	50 (53.2%)		
*p*	26 (27.7%)		
Clinical disease activity, mean (SD)	HBI = 3.9 (3.4)	pMS = 0.8 (1.3)	
Infliximab dose, mg/kg bodyweight, median (range)	8.3 (4.5–11.3)	7.2 (4.1–10.6)	7.9 (4.1–11.3)
Duration of previous infliximab therapy, months, median (range)	30.5 (2–110)	30.0 (3–103)	30.5 (2–110)
Concomitant immunosuppressants, *n* (%)	2 (1.4%)	1 (0.7%)	3 (2.1%)
Concomitant steroids, *n* (%)	0	1 (0.7%)	1 (0.7%)
Concomitant steroid dose, mg/day	0	5	5

BMI, body-mass index; HBI, Harvey–Bradshaw Index; IBD, inflammatory bowel disease; pMS, partial Mayo Score.

### Clinical disease activity

**Figure 2.**
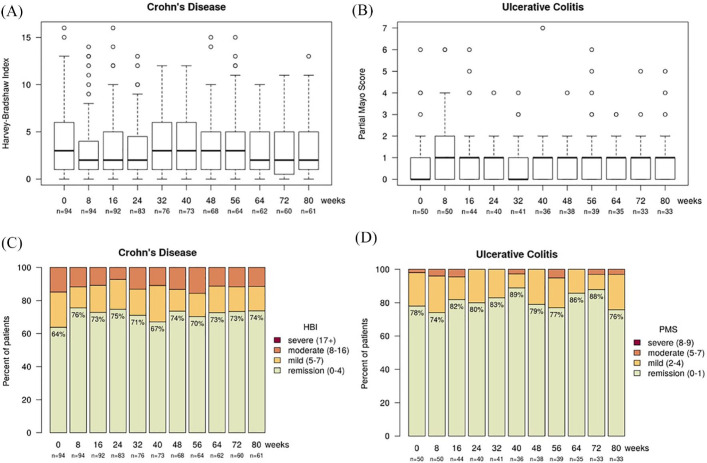
Box blot showing clinical disease activity in (A) Crohn’s disease patients, indicating the median, Q1 and Q3 HBI and in (B) ulcerative colitis patients, indicating the median pMS and the interquartile range. Data are shown for baseline (week 0) and for every 8 weeks over the follow-up period of 80 weeks. Stacked bar plot of clinical disease indices (C) HBI in Crohn’s disease and (D) pMS in ulcerative colitis. The green column indicates patients in clinical remission defined as a HBI ⩽4 or a pMS ⩽1. HBI, Harvey–Bradshaw Index; pMS, partial Mayo Score.

**Figure 3.**
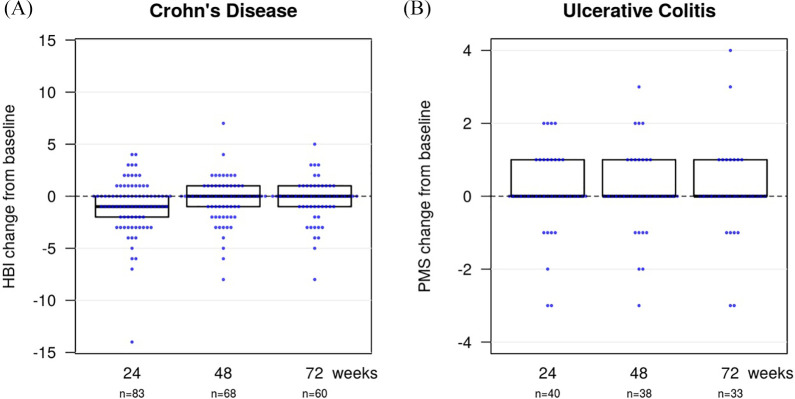
The change in clinical disease activity compared with baseline is shown for (A) Crohn’s disease and (B) ulcerative colitis patients at weeks 24, 48 and 72. HBI, Harvey–Bradshaw Index; pMS, partial Mayo Score.

In CD patients, the median disease activity at baseline (week 0) was a HBI of 3 [interquartile range (IQR) 1–6; *n* = 94]. After switching to the biosimilar SB2, the median HBI was 2 (1–4.5; *n* = 83) at week 24, 3 (1–5; *n* = 68) at week 48 and 2 (0.5–5; *n* = 60) at week 72 (Figure 2). This resulted in a mean change of disease activity (HBI) compared with baseline of –0.9 (SD 2.6) at week 24, –0.4 (2.2) at week 48 and –0.4 (2.0) at week 72 (Figure 3).

In UC patients, the median pMS was 0 (0–1; *n* = 50) at baseline, 1 (0–1; *n* = 40) at week 24, 1 (0–1; *n* = 38) at week 48 and 1 (0–1; *n* = 33) at week 72 (Figure 2). The mean change in the pMS compared with baseline was 0.1 (SD 1.1) at week 24, 0.1 (SD 1.1) at week 48 and 0.1 (SD 1.3) at week 72 (Figure 3).

The proportion of patients in clinical remission at baseline (week 0) was 69% compared with 74% at week 80, respectively. The proportions of patients with mild, moderate and severe disease activity were 21%, 10% and 0% at baseline *versus* 17%, 9% and 0% at week 80. Clinical remission was defined as HBI ⩽4 and pMS ⩽1. Mild disease was defined as HBI of 5–7 or pMS of 2–4, moderate disease as HBI of 8–16 or pMS of 5–7 and severe disease as HBI of >16 or pMS of >7.^22,23^

### CRP levels

**Figure 4.**
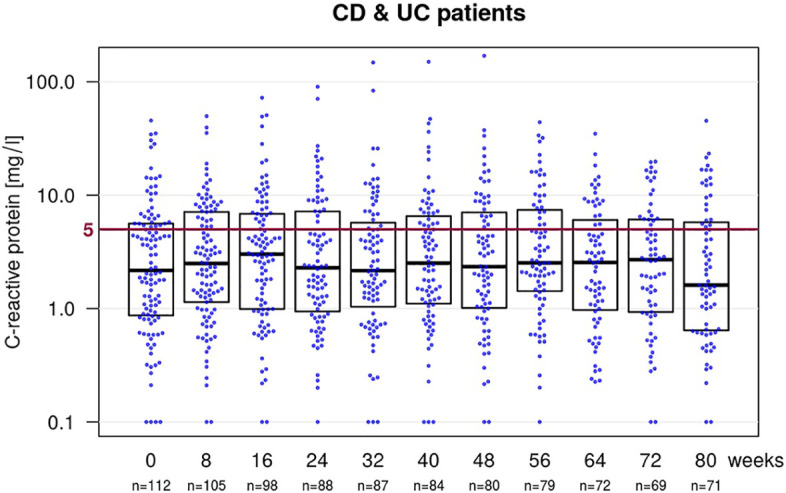
Boxplot with a beeswarm plot showing the levels of C-reactive protein (CRP) at baseline and the follow-up visits for all inflammatory bowel disease patients. CRP levels below 5 mg/l (below the red line) are considered to be normal, as they are below the threshold for positivity in the used test-system. CD, Crohn’s disease; UC, ulcerative colitis.

For CD patients, the median CRP level at baseline (week 0) was 2.5 mg/l (IQR 0.9–5.7; *n* = 71), 1.9 mg/l (0.9–5.6; *n* = 59) at week 24, 2.6 mg/l (1.3–7.5; *n* = 50) at week 48 and 2.8 mg/l (0.9–6.5; *n* = 42) at week 72 (Figure 4), resulting in a median change of 0.5 mg/l (IQR –1.5–2.1 mg/l) at week 72 compared with baseline CRP. For UC patients, the median CRP level at baseline was 1.8 mg/l (0.8–5.6; *n* = 41), 2.4 mg/l (1.2–7.5; *n* = 29) at week 24, 2.2 mg/l (0.6–6.6; *n* = 30) at week 48 and 2.7 mg/l (1.0–4.0; *n* = 27) at week 72, resulting in a median change of 0.0 mg/l (–0.4 to 1.2) at week 72 compared with baseline. For all IBD patients, the median CRP was 2.2 mg/l (0.9–5.6; *n* = 112) at baseline, 2.3 mg/l (0.9–7.2; *n* = 88) at week 24, 2.3 mg/l (1.0–7.1; *n* = 80) at week 48 and 2.7 mg/l (0.9–6.1; *n* = 69) at week 72 and the median change compared with baseline 0.4 mg/l (–0.6 to 1.7) at week 72.

### Pharmacokinetics and immunogenicity

#### TLs

**Figure 5.**
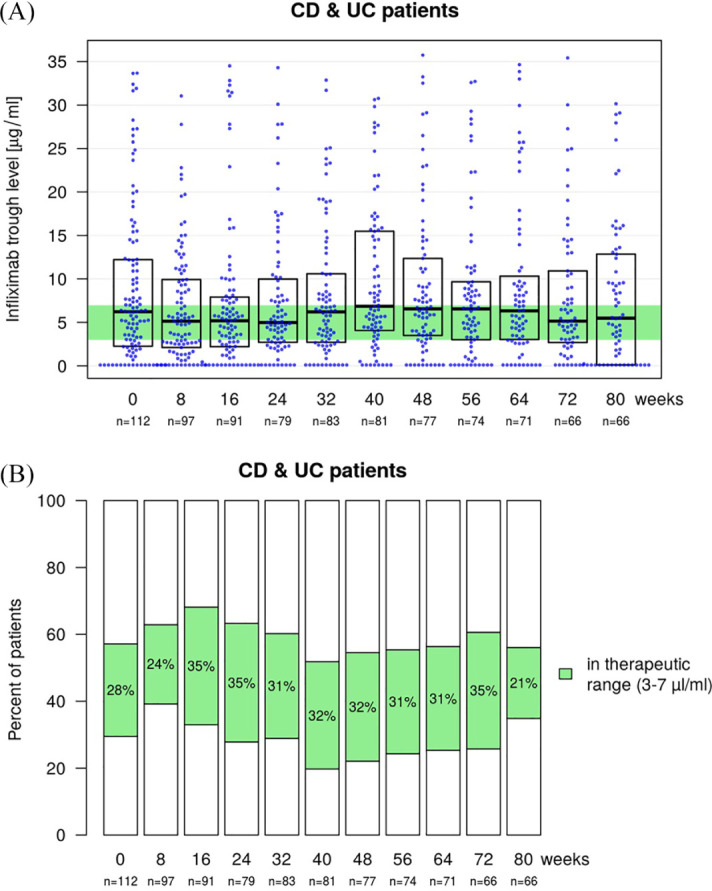
Boxplot with a beeswarm plot (A) showing infliximab trough levels (TLs) at baseline (week 0) and during the 8-week follow-up visits for all inflammatory bowel disease patients. The baseline TLs prior to the first SB2 application refer to originator infliximab. The considered therapeutic range of 3–7 µl/ml is highlighted in green. Stacked bar plot (B) showing the proportion of patients above, within (green) and below the defined therapeutic TL at baseline (week 0) and during the follow-up period. CD, Crohn’s disease; UC, ulcerative colitis.

The median TL at baseline for all IBD patients was 6.2 µg/ml (IQR 2.3–12.2; *n* = 112) referring to originator infliximab before subsequently switching to SB2. After switching to SB2 the median TLs were 5.0 µg/ml (2.7–10.0; *n* = 79) at week 24, 6.6 µg/ml (3.5–12.4; *n* = 77) at week 48 and 5.1 (2.7–10.9; n = 66) at week 72 (Figure 5). This resulted in a median change of TLs compared with baseline of 0.0 µg/ml (–2.8 to 1.5) at week 24, 0.0 µg/ml (–2.4 to 4.2) at week 48 and 0.0 µg/ml (–3.1 to 3.6) at week 72.

The median TL for CD patients at baseline was 6.4 µg/ml (2.7–12.2; *n* = 71) with a median change of TL compared with baseline of 0.0 µg/ml (–2.8 to 1.7) at week 24, 0.0 µg/ml (–2.7 to 4.7) at week 48 and 0.0 µg/ml (–2.8 to 4.6) at week 72. For UC patients, the median TL was 5.7 µg/ml (2.0–10.1; *n* = 41) at baseline with a median change of the TL of –0.1 µg/ml (–2.9 to 1.3) at week 24, 0.0 µg/ml (–2.4 to 2.5) at week 48 and 0.0 µg/ml (–3.0 to 1.8) at week 72 in UC patients. For the subset of patients that had data available for the complete study phase of 80 weeks (*n* = 66), the median TLs were 6.3 µg/ml (2.5–12.2) at baseline, 4.9 µg/ml (2.4–8.0) at week 24, 6.8 µg/ml (3.5–13.2) at week 48 and 5.2 µg/ml (3.1–10.6) at week 72. No clinically significant changes in the median TLs were detected.

The proportion of patients within the defined therapeutic TL range of 3–7 µg/ml were 28% at baseline, 35% at week 24, 32% at week 48 and 35% at week 72, respectively. Patients below and above the therapeutic TL range were 29% and 43% at baseline, 28% and 37% at week 24, 22% and 45% at week 48, and 26% and 39% at week 72 (Figure 5). The results were similar in both patient groups.

#### ADAs

**Figure 6.**
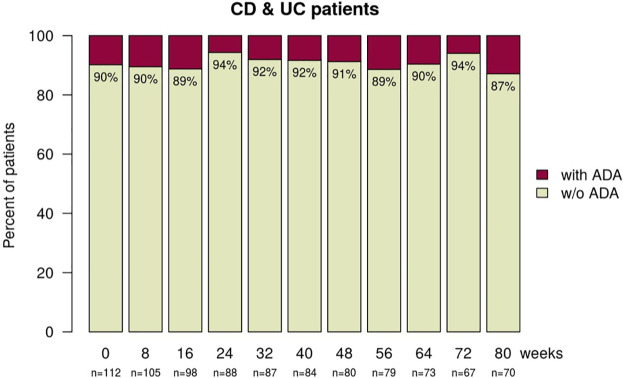
Stacked bar plot showing the proportion of patients with (red) and without (green) ADA at baseline (week 0) and during the follow-up visits for all inflammatory bowel disease patients. ADA, anti-drug antibody; CD, Crohn’s disease; UC, ulcerative colitis.

Pre-existing ADAs at baseline (week 0) were found in 11 (9.8%) patients and persisted in five (4.5%) patients (Figure 6). All five patients with persisting ADAs showed sub-therapeutic TLs. Due to infusion reactions, two patients with pre-existing ADAs discontinued infliximab treatment after the second SB2 infusion. Four patients with pre-existing ADAs lost positivity after switching, resulting in rising TLs for three patients. New and persistent ADAs were observed in seven (6.3%) patients, of which one discontinued infliximab treatment for clinical secondary loss of response (SLR) and one for sustained infusion reactions. New and transient ADAs were detected in four (3.6%) patients at one time-point only. One of these patients received ongoing prophylaxis with prednisolone and clemastine for prophylaxis against an infusion reaction prior to the switch. One patient developed a novel infusion reaction and lost ADA positivity while receiving prophylaxis with prednisolone plus clemastine.

In summary, a minor proportion of patients showed ADA-positivity at baseline and the rate of ADA development after the switch to biosimilar infliximab SB2 was similarly low and not outside the expected range.^24,25^

### Safety

Altogether, 11 serious and 33 non-serious AEs were registered in 40 (27.8%) patients, resulting in a proportion of 7.6% patients with SAE, 20.1% AE and an incidence rate of 5.9 SAE and 17.8 AE in 100 person-years.

There were four malignancies that were diagnosed after switching to SB2: one mammary and one prostate carcinoma, one neuroendocrine tumor and one patient developed chronic lymphatic leukemia (CLL). All patients diagnosed with a malignancy discontinued infliximab treatment, except for the CLL patient. Due to persistent disease activity, surgeries had to be performed in four patients (all CD) and in one patient due to spontaneous sigmoid perforation (UC). One patient developed a liver abscess (CD), and one had to be hospitalized for severe bronchopulmonary infection.

Non-severe events (AEs) referred to infectious complications (10), abscesses (four), newly occurring fistulae (one), infusion reactions (seven), new-onset rheumatoid (five) or dermatologic diseases (three) unrelated to IBD. Mild to moderate infusion reactions appeared in five (3.5%) patients. The majority of patients (4/5) with infusion reactions discontinued infliximab treatment. One patient continued SB2 treatment under prophylaxis. No severe infusion reactions occurred. ADAs were detectable in four out of five patients with infusion reactions.

Concomitant immunosuppressive medication was taken by one patient who developed a liver abscess (azathioprine) and one patient with septic arthritis (prednisolone). All AEs and SAEs are listed in detail in Table 2.

**Table 2.** All (A) serious (B) and non-serious adverse events are listed in this table. Severe adverse events include malignancies, life threatening or potentially disabling conditions and conditions requiring hospitalization. Information about the time point of onset, entity of IBD in the individual and resulting discontinuations of infliximab treatment is provided.

A.				
Patient no.	Serious adverse event	Point of onset (week)	IBD	Discontinuation of IFX therapy
1	Mammary carcinoma	8	UC	Yes
2	Ileocoecal resection	25	CD	Yes
3	Sigmoid stenosis, resection	30	CD	Yes
4	Stenosing anastomisitis, resection	31	CD	Yes
5	Liver abscess	36	CD	Yes
6	Ileus, resection	40	CD	Yes
7	Prostate carcinoma	43	CD	Yes
8	Sigmoid perforation, resection	56	UC	Yes
9	Severe bronchopulmonary infection	57	CD	No
10	Neuroendocrine tumor	75	UC	Yes
11	Chronic lymphatic carcinoma	83	CD	No
B.				
Patient no.	Non-serious adverse event	Point of onset (week)	IBD	Discontinuation of IFX therapy
12	Infusion reaction	0	CD	No
13	Infusion reaction	0	CD	No
	Infusion reaction	8		Yes
14	Infusion reaction	8	CD	Yes
15	Infusion reaction	8	UC	No
	Infusion reaction	16		Yes
16	Infusion reaction	78	UC	Yes
17	Myocarditis following bronchopulmonary infection	9	UC	No
18	Varicella zoster infection	11	CD	No
19	Tinnitus	16	CD	No
20	Polymyalgia rheumatica	16	CD	No
21	Infections (unspecified)	20	CD	Yes
22	Perianal abscess	23	CD	No
	Perianal abscess	42		No
23	Ankylosing spondylitis	28	CD	Yes, but for SLR
24	Banding of esophageal varices in non-alcoholic steatohepatitis	30	CD	No
25	Cutaneous small-vessel vasculitis	31	UC	No
26	Suspected lupus like syndrome	32	CD	Yes, but for SLR
27	*Clostridoides difficile* infection	39	CD	No
	*Clostridoides difficile* infection	62		Yes
28	Exacerbation of psoriasis	42	UC	No
29	Exacerbation of psoriasis	43	UC	No
30	Herpes genitalis	44	UC	No
31	Exanthema	45	CD	Yes
32	Infectious exacerbation of chronic obstructive pulmonary disease	46	CD	No
33	Sigma stenosis	47	CD	No
34	Septic arthritis	48	CD	Yes
35	Benign retroperitoneal tumor	50	CD	No
36	Recurrent vaginal infections	54	CD	Yes
37	Labial abscess	60	CD	No
38	Dermal abscess	64	UC	No
39	Campylobacter enteritis	79	UC	Yes
40	Perianal fistula	83	CD	No

IBD, inflammatory bowel disease; CD, Crohn’s disease; IFX, infliximab; pMS, partial Mayo Score; SLR, secondary loss of response.

### Dose adjustments and concomitant immunosuppressive medication

Compared with baseline, infliximab dosing at the last patient visit remained unchanged in 84 (58.3%) patients, while 30 (20.8%) received higher and 30 (20.8%) lower dosing of SB2. The infusion interval compared with baseline was unchanged for 84 (58.3%) patients, shortened for 31 (21.5%) and prolonged for 29 (20.1%) patients during the study phase.

A minority of patients received immunosuppressive or steroid co-medication at baseline and over the follow-up period of 80 weeks. Immunosuppressive co-medication for IBD was used by three (2.1%) patients (two azathioprine, one methotrexate) at baseline, of whom two continued the same concomitant therapy until the end of the follow-up period. During our study, an additional five (3.5%) patients commenced concomitant immunosuppressive medication. Two patients received azathioprine (one for IBD, one for cutaneous small vessel vasculitis) and two patients methotrexate (one for psoriasis, one for polymyalgia rheumatica). One patient received methotrexate temporarily for CD. Corticosteroids were used by one patient at baseline and by nine (6.3%) patients (prednisolone dosing 2.5–40 mg per day) during the follow-up period. Steroid therapy was ongoing for two (1.4%) patients at week 80. Half of the patients (5/10) used prednisolone at some time during the follow-up period.

Due to mild to moderate infliximab infusion reactions in the patients’ history, prophylaxis was administered prior to infusion to seven (4.9%) patients at baseline and an additional 11 (7.6%) patients over the follow-up period of 80 weeks. In 13 cases the rationale for administration of prophylaxis was mild to moderate infusion reactions, and in five cases to prevent infusion reactions due to a prolonged infusion interval. For prophylaxis, prednisolone (5/18) or prednisolone and clemastine (13/18) were administered prior to infliximab application. Prophylaxis was ongoing in 11 of 18 cases at the last patient visit.

### Drug persistence

Altogether, 42 (29.2%) patients discontinued infliximab treatment and 10 (6.9%) were lost to follow-up, resulting in a mean follow-up period of 67.0 (SD 25.1) weeks for all patients. Treatment discontinuation took place for clinical SLR in 20 (13.9%) patients, for persistent infusion reactions in four (2.8%) patients, in five (3.5%) patients for infectious complications, in one for occurrence of an exanthema and in nine patients (6.3%) for SAEs. Two patients paused infliximab treatment during pregnancy and one due to remission and were not re-introduced to the drug during the follow-up period. An additional six (4.2%) patients paused infliximab therapy (one during nursing, one during pregnancy, two for infectious complications, one due to a stay abroad, one unknown) and were re-introduced to infliximab during the study phase.

**Figure 7.**
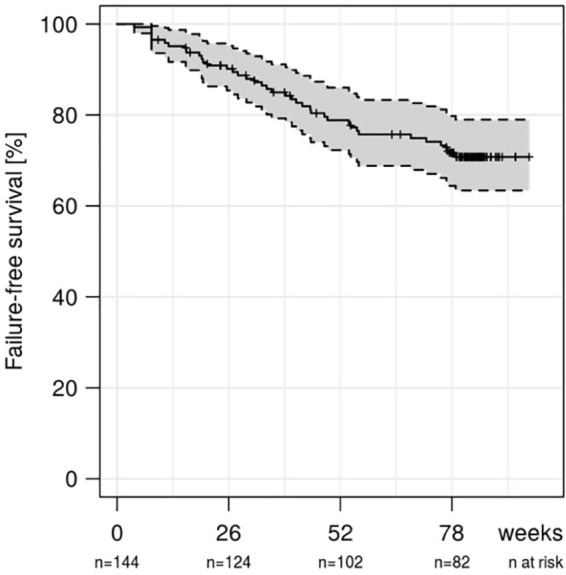
Kaplan–Meier curve showing persistence on infliximab therapy over the follow-up period of 80 weeks for all inflammatory bowel disease patients for informative drop-outs.

All discontinuations of infliximab therapy for SLR, AE, SAE and unknown reasons were defined as informative drop-outs and are shown in Figure 7. For informative drop-outs, persistence on SB2 treatment was 90% (95% confidence interval: 0.85–0.95) at week 26, 79% (0.72–0.86) at week 52 and 72% (0.64–0.80) at week 78.

### Drug acquisition costs

During the study phase of 80 weeks, a total of 10,050 vials (100 mg infliximab/vial) of the biosimilar SB2 were administered. Cumulative cost, based on the manufacturer’s selling price for SB2, was €7,121,198. The manufacturer’s selling price for the same amount of originator infliximab would have been €8,677,689, resulting in a cost difference of €1,556,491 between SB2 and originator infliximab.

## Discussion

This prospective, non-interventional, longitudinal, observational study aimed to evaluate long-term effectiveness, immunogenicity, changes in biomarker CRP levels, and safety following a switch from originator to the infliximab biosimilar SB2 in a real-life setting. While various studies have investigated a switch from originator infliximab to the biosimilar CT-P13, so far there have been published data from only one cohort study of IBD patients in Italy (SPOSIB SB2) regarding treatment with SB2.^5–8,10,16,17^ The analyses included 8 and 17 IBD patients switched from originator infliximab treatment to SB2, with a follow-up time of 6 and 18-months respectively; the majority of patients analyzed in the studied cohort were naïve to infliximab.^16,17^ Based on the study results, overall safety and effectiveness of SB2 were estimated by the authors to be similar to those reported for originator infliximab and CT-P13. Immunogenicity was not assessed in the study. Specific data for patients switched from originator infliximab to SB2 could be found for the 17 IBD patients that were followed-up for 18-months. There was an occurrence of three SAEs in three patients switched from originator infliximab to biosimilar SB2 (17.6%; incidence rate per 100 person-years = 18.9), consisting of one infusion reaction and two infections. At the end of the study, 41.2% of these patients needed dose optimization and two patients (11.8%) had interrupted SB2 treatment.^17^ There were no further data that could be derived from this group of patients, as clinical effectiveness, immunogenicity and change in inflammatory biomarkers following a switch from originator infliximab to the biosimilar SB2 were not studied.

In our IBD cohort, the primary outcome measure of our study, change in clinical disease activity referring to the HBI in CD and the pMS in UC patients, was not affected by the transition to SB2. The mean change of disease activity score compared with baseline was a HBI of –0.4 (SD 2.0) and a pMS of 0.1 (1.3) at week 72, resulting in a proportion of patients in clinical remission (HBI ⩽4, pMS ⩽1) of 69% at baseline and 74% at week 72 for all IBD patients. These data indicate that switching to SB2 was not associated with lack of effectiveness. Our data are in line with the observations made in the NOR-SWITCH trial that showed non-inferiority of the biosimilar CT-P13 to originator infliximab in a cohort of 248 IBD patients (155 CD, 93 UC) for clinical disease activity, as disease worsening occurred in 26% of patients in the originator infliximab group *versus* 30% in the CT-P13 group at week 52.^5^ All patients of the NOR-SWITCH cohort were in stable remission before the switch for a minimum of 6 months under originator infliximab treatment, which is in contrast to our study. Although remission was not a requirement for inclusion, due to the real-life setting of our study, 69% of patients were in remission at the time of enrollment.

The secondary outcome measure, change in CRP levels compared with baseline (0.4 mg/l for week 72), was not compromised by the switch to the biosimilar SB2 in our study, as the median CRP level remained within the normal threshold.

The median TLs were stable during the study phase (6.2 µg/ml at baseline versus 5.1 µg/ml at week 72) for all IBD patients, with a median change compared with baseline of 0.0 µg/ml (IQR –3.1 to 3.6) at week 72, and a consistent proportion of patients within the considered therapeutic range of 3–7 µg/ml (28% at baseline versus 35% at week 72). Higher dosing was not generally required in our cohort.

Pre-existing ADAs were detected in 11 (9.8%) patients, persisted in five, vanished in four and led to discontinuation of SB2 therapy due to infusion reactions in two patients. Immunogenicity remained low with 11 (9.8%) patients developing new ADAs between switch to SB2 and the end of the study, which were in line with previously published rates.^24,25^ Newly developed and persisting ADAs were detected in seven (6.3%), and transient ADAs were detected in an additional four (3.6%) patients at one time-point each. These findings are in line with published data that showed cross-reactivity of ADA to originator infliximab and the biosimilars CT-P13 and SB2 in IBD patients, suggesting full-interchangeability regarding immunogenicity.^9^

Altogether, 42 patients (29.2%) discontinued SB2 treatment and 10 (6.9%) were lost to follow-up. Our data are comparable to the findings in the SPOSIB SB2 cohort, in which 26.1% of all patients interrupted SB2 treatment during the same follow-up time of 18 months.^17^ The 13.8% SB2 treatment discontinuation rate due to SLR over 18 months in our cohort is also within the expected range, as the annual risk for loss of infliximab response was calculated to be 13% per patient-year.^26^ The incidence rates in this IBD cohort were 5.9 per 100 person-years for SAE and 17.8 per 100 person-years for AE. There were no unexpected safety signals. Compared with the SPOSIB SB2 cohort (36.7 SAE per 100 person-years) the incidence rate remained relatively low in our cohort.^17^ Our data regarding effectiveness and safety are in line with the available results for switching procedures from originator infliximab to the biosimilar CT-P13.^5–8,10^ There was a price difference of €1,556,491 between SB2 and originator infliximab in terms of drug acquisition costs, resulting in substantial health-care cost savings.

Our study has several limitations which can mainly be attributed to its real-life setting. First, there was no internal control group of patients continuing originator infliximab treatment, as all patients on originator infliximab were switched to SB2. Therefore, our SB2 cohort could not be compared with a contemporary infliximab originator regarding effectiveness (equivalence), immunogenicity and safety. Second, the cohort was heterogeneous, consisting of patients with a wide range of differing durations of previous infliximab treatment, disease activities and durations, infliximab dosing and infusion intervals. Endoscopic evaluation could not be integrated into our study to assess mucosal inflammatory activity at baseline and during the follow-up period. Furthermore, fecal calprotectin, an established surrogate marker for endoscopic disease severity, could not be assessed in our study setting. The described effectiveness of biosimilar SB2 treatment therefore needs to be interpreted cautiously. We were, however, able to regularly assess CRP levels in each patient. Although CRP levels have been shown to be a poor surrogate marker for endoscopic disease activity, normalization of CRP levels as therapeutic response has been shown to predict relapse-free survival and prevent need for surgery in UC.^27–30^ Correspondingly, only one UC and four CD patients had to undergo a surgical procedure in our examined group of patients.

The strengths of this prospective study are the large cohort of IBD patients with a high density of data systematically collected at every patient visit. Furthermore, this is the first study investigating long-term clinical effectiveness in an IBD cohort following a switch from originator infliximab to SB2, additionally providing data on pharmacokinetics, immunogenicity, biomarkers and safety, thereby enabling profound insights into the outcomes of switching to SB2. Regarding multiple switches between originator infliximab and infliximab biosimilars, further investigations will be necessary.

Based on the presented results we want to conclude that a switch from originator infliximab to the biosimilar SB2 was not associated with a medically meaningful change in clinical disease activity, CRP levels, change in the pharmacological profile or immunogenicity over the follow-up period of 80 weeks. Furthermore, the switch to SB2 treatment was well tolerated and there was no increased incidence of AEs or SAEs related to SB2, indicating that switching from originator infliximab to SB2 represents a feasible option in IBD patients.
